# Person-centered contraceptive counseling and associations with contraceptive practices among a nationally representative sample of women in Ethiopia^☆^

**DOI:** 10.1016/j.conx.2024.100114

**Published:** 2024-11-19

**Authors:** Celia Karp, Shira Tikofsky, Solomon Shiferaw, Assefa Seme, Mahari Yihdego, Linnea Zimmerman

**Affiliations:** a Department of Population, Family and Reproductive Health, Johns Hopkins Bloomberg School of Public Health, Baltimore, MD, USA; b Department of Reproductive Health and Health Service Management, School of Public Health, Addis Ababa University, Addis Ababa, Ethiopia

**Keywords:** Counseling, Contraception, Ethiopia, Family planning, Person-centeredness, Quality of care

## Abstract

**Objectives:**

To estimate levels of person-centered contraceptive counseling among current and recent contraceptive users, assess for whom counseling differs, and examine the relationship between counseling and contraceptive practices, specifically use of provider-dependent methods and use of one’s preferred method, among women in Ethiopia.

**Study design:**

This cross-sectional study uses nationally representative data collected by the Performance Monitoring for Action Ethiopia project among current and recent contraceptive users (*n* = 2731) aged 15–49 between October and November 2021. Descriptive analyses estimated person-centered counseling levels via the recently validated quality of contraceptive counseling short scale (QCC-10). Bivariable and multivariable logistic regression estimated associations with contraceptive practices.

**Results:**

Contraceptive users in Ethiopia receive moderate quality counseling (mean QCC-10 score = 2.69, range: 1.1–4.0) with significant social inequities in the receipt of person-centered care. Women who are younger, uneducated, not in union, from poorer households, or who sourced their method from a non-public facility reported less person-centered care. Strong relationships were observed between higher quality counseling and women’s contraceptive practices. Those receiving highest quality counseling had nearly double the odds of using provider-dependent methods compared to those reporting lowest quality counseling (AOR: 1.92; 95% CI: 1.16–3.18). Among current users, women reporting highest quality counseling had 62% higher odds of using their preferred method relative to women receiving poorest quality care (95% CI: 1.06–2.48).

**Conclusion:**

Poorer quality care is associated with use of non-preferred methods and reliance on provider-independent methods. Efforts to reduce reproductive health disparities and promote contraceptive autonomy should prioritize a person-centered approach to contraceptive counseling for all.

**Implications:**

Inequitable delivery of person-centered contraceptive care based on individuals’ sociodemographic characteristics, such as education or marital status, undermines women’s reproductive autonomy and hinders contraceptive experiences. Person-centered contraceptive counseling should be provided to all women in Ethiopia, regardless of their background, to support individuals in achieving their reproductive goals.

## 1 Introduction

Counseling is the cornerstone of family planning services, ensuring individuals receive comprehensive and accurate information about their contraceptive options. Person-centeredness is a key dimension of counseling quality, encompassing care that is tailored to individuals’ unique preferences, needs, and values. This approach emphasizes personalized information exchange, supportive interpersonal relations, and respectful treatment, recognizing that reproductive decisions are deeply personal and must be guided by patient values [1]. Person-centered care is essential to protecting individuals’ rights to make informed and unrestricted decisions about their reproductive health. People who receive higher quality, person-centered counseling are more likely to continue using their method by choice, have their informational needs about contraception met, and exercise greater autonomy in contraceptive decisions [1], [2], [3], [4].

Quality of contraceptive care is a particularly salient issue in Ethiopia. Evidence from 2019 indicates only 12% of contraceptive users reported being told about other methods, side effects of their method, and what to do if they experienced side effects, as measured by the Method Information Index (MII), and this proportion declined by more than half since 2015 [5]. Counseling about side effects also varies by contraceptive method type and the type of facility from which people source methods, highlighting inequities in care [6]. Experiences of poorer quality contraceptive care may be due to a myriad of reasons, including commodity availability, inadequately trained and/or overworked staff, or provider bias, resulting in discrimination against specific populations (e.g., unmarried, young women) or the promotion of one method type (e.g., provider-dependent methods, such as implants) over others [7], [8], [9], [10].

In 2021, the Ethiopian Federal Ministry of Health (FMOH) identified the need for “quality and comprehensive rights-based family planning” as a key tenant of their five-year health sector transformation plan [11]. This priority was further reflected in national family planning guidelines, which emphasized the need for high-quality contraceptive care that is compassionate, respectful, and client-centered [12]. Despite this focus, measurement of rights-based and person-centered care in Ethiopia is limited by current indicators, many of which fail to address how individuals’ needs and preferences are met in contraceptive counseling interactions [2], [13], [14], [15].

Reliance on proxy measures of quality, like the MII and the expanded MII Plus (asking about counseling on method switching), neglects key aspects of care experiences, such as interpersonal treatment, respect for informed choice, and experience of coercion or bias. The recently developed Quality of Contraceptive Counseling scale (QCC)—and its adapted, 10-item short scale, the QCC-10—adopts a person-centered and human rights framework to evaluate the degree to which individuals’ contraceptive care experiences align with their unique needs and preferences [1], [2], [16], [17]. Complementing other measures of quality in contraceptive counseling, the QCC-10 emerged from robust, formative research and cognitive testing to align scale items with individuals’ experiences of care and capture domains of person-centeredness that have traditionally been excluded from measurement (e.g., disrespect and abuse, privacy, confidentiality). The original QCC scale was developed for use in Mexico and later refined and validated in Burkina Faso, Ethiopia, India, Kenya, and Nigeria [2], [17], [18]. This new measure of person-centered contraceptive counseling, offering nuance in assessing experiences of contraceptive care with direct measurement of negative interactions and personal preferences, values, and needs, can aid in monitoring progress toward the FMOH’s goal of enhancing quality and delivery of rights-based contraceptive services. While validated for use in the Ethiopian context, the QCC-10 has yet to be used for understanding experiences of contraceptive counseling and inequities in care at the national level in Ethiopia.

This study uses nationally representative data to (1) estimate levels of person-centered contraceptive counseling using the QCC-10 scale among current and recent contraceptive users, (2) assess for whom person-centered counseling differs, and (3) examine the relationship between person-centered counseling and contraceptive practices, specifically use of provider-dependent methods and use of preferred methods, among women in Ethiopia.

## 2 Methods

### 2.1 Study design

This cross-sectional study uses nationally representative survey data collected among women aged 15–49 between October and December 2021 by the Performance Monitoring for Action (PMA) Ethiopia study, a collaboration between Addis Ababa University, FMOH, and Johns Hopkins University [19]. Multistage sampling using probability proportional to size within six regions of Ethiopia, which cover approximately 90% of the country’s population: Addis Ababa, Afar, Amhara, Oromia, Southern Nations, Nationalities, and Peoples’ Region (SNNP-R), and Tigray, and urban/rural strata was used to select 265 enumeration areas (EAs). These regions were selected as they cover the majority of the population in Ethiopia and due to their diverse sociodemographic, ethnic, and health system characteristics; with wealthier populations and better healthcare access in the capital, Addis Ababa, compared to the higher proportion of rural, largely agrarian residents of Amhara, SNNP-R, Oromia and Tigray, and finally Afar, which is unique in that it remains a largely pastoralist region, with the lowest access to care. Thirty-five households were randomly selected from a complete household listing within each EA. All women aged 15–49 years who were either usual members of the household or who slept in the household the night before were eligible for survey participation and verbal informed consent was obtained from participants. Study procedures were approved by Addis Ababa University [075/13/SPH] and Johns Hopkins Bloomberg School of Public Health [FWA00000287] Institutional Review Boards. Additional information about PMA Ethiopia procedures, including detailed information on consent processes, is described elsewhere [19].

### 2.2 Measures

**Fig. 1:**
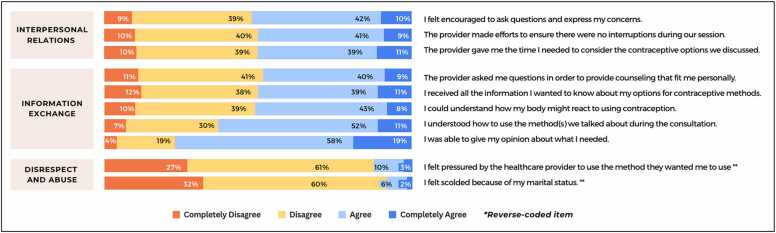
Distributions of women’s responses to each QCC-10 item, among a nationally representative sample of current and recent contraceptive users in Ethiopia, 2021 (*N* = 2731). QCC-10 = quality of contraceptive counseling short scale.

The primary outcome was person-centeredness in contraceptive care, measured via the QCC-10, a validated tool for use in Ethiopia [16], [18]. Adapted from the longer 22-to-25 item QCC scale, the QCC-10 measures three domains of quality—*information exchange* (five items), *interpersonal relations* (three items), and *disrespect and abuse* (two items) [1], [18] (Fig. 1). Women indicated their level of agreement with each QCC-10 item based on their experience receiving their current or most recent contraceptive method. Response options ranged from “completely agree” to “completely disagree” and were coded on a four-point Likert scale (range: 1–4) [20]. Two items measuring disrespect and abuse were reverse coded for consistency; higher scores indicated higher quality counseling. In addition to item-specific responses, composite scores were calculated using an average of all item responses (range: 1–4) in alignment with the original measure development and for ease of interpretation and use within health facilities or family planning programs. Additionally, a categorical measure was developed by evenly dividing continuous QCC-10 scores into tertiles (lowest, middle, highest).

Secondary outcomes included contraceptive method type (grouped as provider-dependent vs. provider-independent) and, among current users, use of one’s preferred method (yes/no). Provider-dependent methods were those that required interactions with healthcare providers for their initiation and/or discontinuation, including sterilization, intrauterine devices (IUDs), implants, and injectables, while provider-independent methods were pills, emergency contraceptives, male/female condoms, and standard days/cycle beads, most of which could be sourced from lower cadre healthcare providers or lower level facilities. We explored women’s use of a provider-dependent method to assess how provider interactions—measured via the QCC-10—related to use of methods requiring ongoing provider involvement. We hypothesized that women receiving lower quality care may be less likely to adopt methods that require re-engagement with providers for discontinuation. Sociodemographic characteristics included age, partnership status (in-union vs. not), education (none, primary, secondary or and higher), wealth quintile (calculated by the project during dataset creation via principal components analysis at the household level based on assets owned and household construction materials, with scores then grouped into quintiles), residence (urban vs. rural), parity, and region. Contraceptive characteristics included current contraceptive use status (current/recent user) and where the method was sourced (public higher-level facility [e.g., hospital, health center], health post/Health Extension Worker [HEW], or non-public facility).

### 2.3 Analysis

Descriptive statistics summarized sociodemographic and contraceptive characteristics, in addition to distributions of responses to each QCC-10 item and composite QCC-10 scores. Psychometric analysis examined internal consistency reliability of the QCC-10 scale using Cronbach's alpha [20], [21]. Bivariate and multivariate linear regression models tested for mean differences in QCC-10 composite scores according to women’s sociodemographic contraceptive characteristics. Next, separate bivariate and multivariate logistic regression models estimated associations between composite QCC-10 scores and (1) method type used and (2) use of one’s preferred method, examining differences among women who received the lowest, middle, and highest quality counseling for ease of interpretation. Multivariate models adjusted for sociodemographic and contraceptive characteristics (i.e., current use status, method source) as potential confounders. Survey weights adjusted for the complex survey design.

### 2.4 Analytic sample

Altogether, 8070 women aged 15–49 completed the cross-sectional survey. We restricted analysis to those who were sexually active, current or recent (<2 years) users of modern contraception (*n* = 2731), and further limited secondary analysis of preferred method use to current users (*n* = 1883). No women from Somali were current/recent users, thereby excluding this region from analysis.

## 3 Results

Nearly half of women were aged 20–29 years and most were married/in-union (Table 1). Two-thirds were currently using contraception, with injectables accounting for more than half of all current and recent contraceptive use. Roughly half of women sourced their current or most recent method from a public higher-level health facility (i.e. government-operated hospitals, health centers, pharmacies, and other government-operated health facilities).

**Table 1:** Weighted sociodemographic and contraceptive characteristics among a nationally representative sample of current and recent contraceptive users in Ethiopia, 2021 (*N* = 2731)

Characteristic	*N* (%)
Age	
15−19	182 (6.1)
20−29	1436 (48.4)
30−39	1025 (34.5)
40−49	327 (11.0)
Partnership Status	
Not in union	251 (9.5)
In-union	2718 (91.5)
Parity	
0	402 (13.5)
1	687 (23.2)
2−3	996 (33.6)
4 or more	882 (29.7)
Education	
None	858 (28.9)
Primary	1276 (42.9)
Secondary or higher	835 (28.1)
Wealth^a^	
Poorest	465 (15.7)
Lower	574 (19.3)
Middle	549 (18.5)
Higher	569 (19.2)
Residence	
Urban	1033 (34.8)
Rural	1937 (65.2)
Region	
Afar, BG, DD, Gambella, Harari^b^	83 (2.8)
Amhara	878 (29.5)
Oromia	1259 (42.4)
SNNP-R	364 (12.3)
Addis Ababa	225 (7.6)
Sidama	161 (5.4)
Contraceptive Use	
Current user	1995 (67.2)
Recent user (within last 2 y)	974 (32.8)
Method Type Used	
Provider-dependent	
Implant	678 (22.8)
Intrauterine device (IUD)	44 (1.5)
Injectables	1725 (58.1)
Female Sterilization	33 (1.1)
Provider-independent	
Pill	271 (9.1)
Emergency Contraceptive	38 (1.3)
Male Condoms	24 (0.8)
Std. Days/Cycle Beads	8 (0.3)
Method Source	
Public non-health post^c^	1405 (49.5)
Health post/HEW^d^	627 (22.1)
Non-public facility	806 (28.4)

a Wealth quintile assigned based on distribution of household wealth scores, which are generated using principal components analysis of household assets and construction materials.

b Region includes Afar, Benishangul Gumuz, Dire Dawa, Gambella, Harari; combined due to small samples. SNNP-R = Southern Nations, Nationalities, and Peoples' Region.

c Public non-health post includes government hospitals, health centers, pharmacies, and other government-operated health facilities, except for health posts.

d HEW = health extension worker.

Distributions of responses varied by quality domain (Fig. 1). In the domain of interpersonal relations, relatively balanced proportions of women agreed and disagreed with most items. For example, half of women agreed/completely agreed that the provider tried to ensure privacy (i.e., prevent interruptions) and allocated sufficient time for contraceptive decision-making (50% and 51%, respectively). Similar patterns were observed for information exchange items. Higher proportions of women endorsed statements regarding their understanding of how to use their method and their ability to express their opinions in the consultation (63% and 77% of women agreed/completely agreed, respectively). A minority (8–13%) reported some form of disrespect or abuse.

**Fig. 2:**
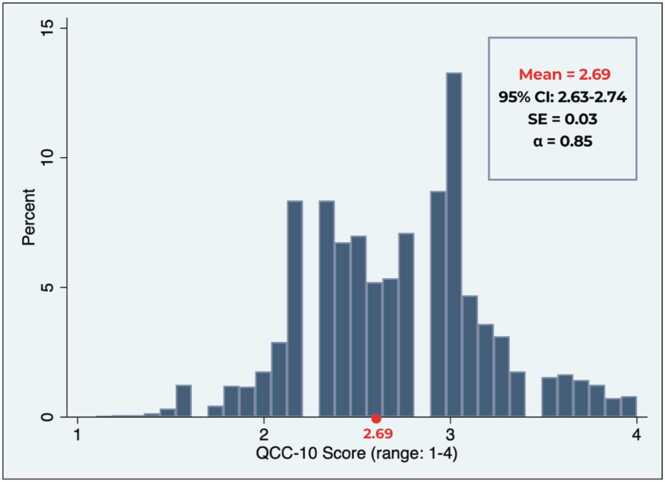
Distribution of QCC-10 composite score among a nationally representative sample of current and recent contraceptive users in Ethiopia, 2021 (*N* = 2731). QCC-10 = quality of contraceptive counseling short scale.

Women’s composite QCC-10 scores ranged from 1.1 to 4.0 (mean = 2.69, 95% CI: 2.63–2.74), with lower scores indicating poor quality and less person-centered care. On average, results suggested a moderate level of quality counseling among women in Ethiopia (Fig. 2). Cronbach’s alpha was 0.85, indicating high internal consistency reliability. In unadjusted models, higher QCC-10 scores were observed among women who were aged 20–29, in-union, attended primary or higher education levels, wealthier, or currently using contraception (Table 2). Similarly, regional and facility-based variation in person-centered contraceptive care was observed with higher QCC-10 scores among those residing in the combined smaller regions (i.e., Afar, Benishangul Gumuz, Dire Dawa, Gambella, Harari), Oromiya, or Addis Ababa, relative to Amhara, while women sourcing their method from health posts/HEWs or non-public facilities reported lower scores (Table 2). Results remained largely consistent in adjusted models, though residing in Oromiya or sourcing contraception from a health post/HEW was no longer linked to poorer quality. Women aged 30–39 and 40–49 had 0.11-point (95% CI: 0.02–0.21) and 0.13-point (95% CI: 0.01–0.25) higher QCC-10 scores, respectively, compared to adolescents aged 15–19, while those in-union had a 0.11-unit increase in QCC-10 scores relative to those not in-union (95% CI: 0.03–0.20). Sourcing one’s method from a non-public facility, such as a private clinic/pharmacy, was associated with 0.21-point (95% CI: −0.30 to −0.17) lower QCC-10 scores, relative to women who received care from higher-level public facilities.

**Table 2:** QCC-10 composite score by sociodemographic and contraceptive characteristics among a nationally representative sample of current and recent contraceptive users in Ethiopia, 2021 (*N* = 2731)

Characteristics	Unadjusted Mean Difference (95% CI)	*p*-value	Adjusted Mean Difference^a^ (95% CI)	*p*-value
Age (Ref: [15], [16], [17], [18], [20])				
20−29	**0.11 (0.11–0.21)**	**0.03**	0.06 (−0.09 to 0.10)	0.13
30−39	0.12 (0.01–0.23)	0.05	**0.11 (0.02–0.21)**	**0.02**
40−49	0.12 (−0.11 to 0.26)	0.07	**0.13 (0.01–0.25)**	**0.04**
Partnership (Ref: Not in-union)				
In-Union	**0.14 (0.06–0.21)**	**<0.01**	**0.11 (0.03–0.20)**	**<0.01**
Parity (Ref: Nulliparous)				
1	0.07 (−0.02 to 0.16)	0.13		
2−3	0.01 (0.01–0.18)	2.21		
4 or more	0.18 (−0.06 to 0.10)	0.43		
Education (Ref: None)				
Primary	**0.07 (0.01–0.13)**	**0.02**	**0.07 (0.01–0.13)**	**0.02**
Secondary or higher	**0.18 (0.09–0.26)**	**<0.01**	**0.15 (0.06–0.23)**	**<0.01**
Wealth (Ref: Lowest)				
Lower	0.52 (−0.03 to 0.14)	0.21	0.04 (−0.05 to 0.12)	0.46
Middle	0.07 (−0.16 to 0.16)	0.11	0.07 (−0.03 to 0.16)	0.13
Higher	**0.14 (0.13–0.25)**	**0.01**	0.11 (−0.01 to 0.23)	0.06
Highest	**0.18 (0.07–0.29)**	**<0.01**	0.12 (−0.01 to 0.24)	0.05
Residence (Ref: Rural)				
Urban	0.97 (−0.01 to 0.19)	0.09		
Region (Ref: Amhara)				
Afar, BG, DD, Gambella, Harari^b^	**0.31 (0.19–0.43)**	**<0.01**	**0.25 (0.13–0.37)**	**<0.01**
Oromiya	**0.14 (0.01–0.27)**	**0.04**	0.13 (0.01–0.26)	0.05
SNNP	0.11 (−0.01 to 0.22)	0.08	0.07 (−0.05 to 0.18)	0.26
Addis Ababa	**0.24 (0.10–0.37)**	**<0.01**	**0.16 (0.01–0.31)**	**0.03**
Sidama	0.06 (−0.07 to 0.19)	0.34	0.03 (−0.10 to 0.17)	0.64
Contraceptive use status (Ref: Recently used)				
Currently using	**0.06 (0.01–0.12)**	**0.03**	0.02 (−0.03 to 0.07)	0.49
Method source (Ref: Public higher-level facility)^c^				
Health post/HEW^d^	**−0.10 (−0.20 to −0.01)**	**0.04**	−0.03 (−0.12 to 0.07)	0.56
Non-public facility	**−0.20 (−0.29 to −0.11)**	**<0.01**	**−0.21 (−0.30 to −0.17)**	**<0.01**

Results from bivariable and multivariable logistic regression models values statistically significant at *p* < 0.05 bolded.

a Model adjusts for age, partnership, education, wealth, region, contraceptive use, and method source. Parity excluded due to collinearity with age. Residence excluded due to collinearity with wealth.

b Region includes Afar, Benishangul Gumuz, Dire Dawa, Gambella, Harari; combined due to small samples. SNNP-R = Southern Nations, Nationalities, and Peoples' Region.

c Public higher-level facility includes government-operated hospitals, health centers, pharmacies, and other government-operated health facilities, except for health posts.

d HEW = health extension worker.

**Fig. 3:**
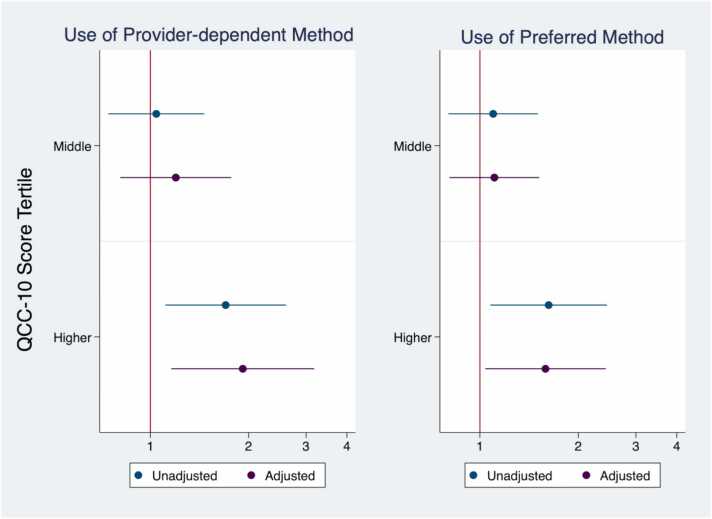
Use of provider-dependent methods and preferred methods by QCC-10 tertile score among a nationally representative sample of current and recent contraceptive users in Ethiopia, 2021. Note: Results from bivariable and multivariable logistic regression models. Use of provider-dependent method assessed among current and recent users. Use of preferred method assessed only among current users. Adjusted for age, partnership, education, wealth, region, contraceptive use status, and method source. QCC-10 = quality of contraceptive counseling short scale.

Women’s contraceptive practices varied according to the quality of care they received (Fig. 3). The proportion of women using provider-dependent methods was highest among those with highest quality counseling (91.5%) compared to those with medium and lower QCC-10 scores (86.9% and 86.4%, respectively; data not shown). In unadjusted models, women who received the highest quality of contraceptive counseling had 70% higher odds of using provider-dependent methods, relative to those receiving lowest quality (95% CI: 1.11–2.61). This relationship was stronger after adjustment for sociodemographic and contraceptive characteristics; women had nearly double the odds of using a provider-dependent methods if they received highest quality counseling, compared to those reporting poorest quality (AOR = 1.92; 95% CI: 1.16–3.18). No such relationship was observed among women who reported moderate, relative to lowest, quality counseling.

Among current contraceptive users, use of one’s preferred contraceptive method was greatest among those reporting highest quality counseling (88.2%), relative to moderate (83.4%) and low (82.1%) quality counseling (data not shown). In unadjusted models, women reporting highest quality counseling had 66% higher odds of using their preferred contraceptive method, relative to those who received lowest quality counseling (95% CI:1.1–2.5; Fig. 3). This positive relationship between quality counseling and preferred method use remained consistent in adjusted models (AOR: 1.62; 95% CI: 1.06–2.48).

## 4 Discussion

Among a nationally representative sample of current and recent contraceptive users in Ethiopia, we find that women in Ethiopia receive moderate levels of person-centered counseling, on average, with significant social and health system inequities in who receives higher quality care. Poorer quality counseling is linked to use of non-preferred methods and reliance on methods that do not require provider involvement. As the first population-based study to examine a validated measure of person-centered contraceptive care in Ethiopia, results reinforce the need to enhance respectful communication, supportive care, and tailored information during family planning visits. In addition, findings highlight implications that this care may have on individuals’ contraceptive practices and ability to achieve their reproductive goals.

The receipt of low-to-moderate quality counseling reported by current and recent contraceptive users (average QCC-10 score = 2.69 on a scale of 1–4) echoes recent evidence demonstrating a lack of information about side effects and method options during family planning visits in Ethiopia [5], [6]. Our results further illustrate how the delivery of person-centered care, including protection of privacy, personalized communication about contraceptive options, and non-discrimination in care, is inadequate for many individuals seeking contraceptive services in Ethiopia. Relative to a recent facility-based study using the original 28-item QCC scale in three regions of Ethiopia [17], we find a lower degree of person-centeredness among our national sample of current and recent users (QCC average = 3.16, range 1–4), though this variation may reflect measurement differences due to our use of the shorter, 10-item measure. Additionally, expansion to a nationally representative sample of women in our study may reveal a broader range of contraceptive care experiences, including poorer quality counseling for women in some regions. A growing focus on ensuring individuals have informed, full, and free choice in contraceptive decisions [20] requires an emphasis on these aspects in counseling interactions to provide individuals with sufficient information and options and support patient autonomy in deciding if and when to use family planning.

We also find significant social and health systems disparities in how person-centered contraceptive care is delivered, with women who are younger, uneducated, or not in union reporting poorer interactions, as well as those who received methods from non-public facilities. Bias in contraceptive services, particularly against younger, unmarried women, and pressure to use specific methods, as reported by 13% of women in our sample, is well-documented [7], [8], [10], [22], reflecting threats to individuals’ reproductive autonomy. Taken together, these results highlight opportunities for enhanced provider-based resources, emphasizing equity in the delivery of contraceptive services, to support the government’s commitment to improving access to high-quality family planning for all [11]. Recognizing that biases in the provision of contraceptive care reflect the systems in which providers operate, social and behavior change approaches, such as values clarification exercises, should be implemented in health facilities to not assign blame at the individual level and, instead, promote supportive contraceptive care environments for all [10]. Our finding that individuals who sourced their contraceptive methods from non-public facilities reported lower quality of care suggests private health sector providers may face distinct challenges to care (e.g., time constraints, limited method options) and may benefit from parallel efforts implemented to improve quality within Ethiopia’s public health system. Results indicate a need for investigation into the applicability of quality metrics across a diversity of contraceptive methods and health facility types, like drug shops/pharmacies (grouped within the non-public facilities in this study) for which the content of counseling interactions may vary considerably.

When examining links between person-centered counseling and contraceptive practices, higher quality care was associated with greater use of provider-dependent methods (e.g., implants, injectables, or IUD) and use of one’s preferred method, relative to women reporting poorest quality care. This is consistent with previous population-based studies in Ethiopia on other aspects of contraceptive counseling, which have found that women who use provider-dependent methods are more likely to be counseled on all aspects of the MII [23]. Additionally, among smaller facility-based samples, women reporting no disrespect or abuse during counseling were more likely to use injectables, relative to no method, compared to those who reported provider pressure to use specific methods or bias against the client [17], [23].

Experiences of respectful, supportive, and tailored counseling may foster trust in health providers and instill a greater willingness among contraceptive users to re-engage with the health system to discontinue their methods, while negative experiences may do the opposite [24], [21]. Alternatively, less person-centered counseling among women using provider-independent methods (e.g., pills, condoms) may be partly attributable to the fact that many women source these methods from pharmacies and receive them with minimal interaction with providers, relative to women seeking long-acting methods from higher-tier facilities. We adjusted for method source (e.g., health post/HEW, non-public facility, etc.) in our multivariable models of method type to reduce potential confounding by facility or provider type. While we anticipated that women using provider-dependent methods might receive more comprehensive counseling on aspects such as the insertion process, pain management, and potential side effects, we did not formulate specific hypotheses regarding the person-centeredness of these interactions, relative to the experiences of women using provider-independent methods. Our findings of inequities in receipt of person-centered counseling by method type used may reflect differences in the content and context in which family planning counseling takes place. Continued investments should be made to promote person-centered counseling across diverse contraceptive delivery channels, regardless of method type, to support individuals’ in achieving their reproductive goals. Research is needed to elucidate differences in how person-centered counseling is delivered in private facilities, mostly pharmacies, where individuals largely source provider-independent methods and identify how client expectations and quality of care in these settings may differ from other contraceptive care interactions.

This study has several limitations. First, data rely on participants’ self-report regarding contraceptive experiences, which may be prone to recall bias, particularly among women no longer using contraception. To address potential recall bias, multivariable models adjusted for current contraceptive use status and sensitivity analyses were run separately by contraceptive use status, with no differences in results. Given that most women in our sample used implants (22.8%) or injectables (58.1%), it also may have been easier to reflect on provider interactions during the visit of method initiation than it would be for individuals who used provider-independent methods, which may be sourced from multiple venues, like pills or condoms. Second, our outcome of preferred method use was only measured among current users, which may exclude those who discontinued their method due to dissatisfaction with a non-preferred method. Our cross-sectional exploration of the relationship between the QCC-10 and preferred method use limited understanding of how person-centered care may shape contraceptive practices over time. As participants were reporting on counseling quality while still using their method, the sample likely had a higher rate of preferred method use than would be found in a longitudinal study, potentially attenuating the observed relationship with the QCC-10.

Despite these limitations, this study has several strengths. We use nationally representative data in Ethiopia and the QCC-10, a recently validated measure of quality in contraceptive care, which explicitly accounts for person-centeredness, human rights, and negative experiences of care in contraceptive services, thereby expanding a body of literature focused largely on the content of counseling interactions via the MII and MII Plus. In addition, this study examines links between counseling experiences and contraceptive practices to elucidate these relationships. Future research may use the QCC-10, or individual items reflecting key aspects of counseling, to understand how counseling experiences relate to reproductive practices over time and develop actionable recommendations for improving the delivery of high-quality, person-centered contraceptive services in Ethiopia.

## Author contributions

S.S.: Writing – review & editing, Project administration, Funding acquisition, Data curation. A.S.: Writing – review & editing, Project administration, Funding acquisition, Data curation. C.K.: Writing – review & editing, Writing – original draft, Visualization, Validation, Methodology, Investigation, Formal analysis, Data curation, Conceptualization. S.T.: Writing – review & editing, Formal analysis. M.Y.: Writing – review & editing, Project administration, Data curation. L.Z.: Writing – review & editing, Project administration, Methodology, Funding acquisition, Data curation, Conceptualization.

## Declaration of Competing Interest

The authors declare no competing interests.
